# Skin-Derived Non-Coding RNAs as Potential Biomarkers and Therapeutic Targets

**DOI:** 10.3390/ncrna12050038

**Published:** 2026-09-28

**Authors:** Jane L. Zen, Quynh Mai Nguyen Le, Shizhou Li, George B. Hanna

**Affiliations:** Department of Surgery and Cancer, Imperial College London, Hammersmith Hospital, London W12 0NN, UK; quynh.nguyen-le23@imperial.ac.uk (Q.M.N.L.); shizhou.li22@imperial.ac.uk (S.L.); g.hanna@imperial.ac.uk (G.B.H.)

**Keywords:** non-coding RNA, microRNA, long non-coding RNA, circular RNA, skin biomarkers, sweat, interstitial fluid, liquid biopsy

## Abstract

Skin-derived extracellular nucleic acids—particularly non-coding RNAs (ncRNAs) such as miRNAs and lncRNAs—are a rapidly evolving frontier in molecular diagnostics. These molecules are present in skin tissue, dermal interstitial fluid, and sweat, whilst ncRNAs regulate essential processes including epidermal differentiation, wound healing, immune regulation, and tumourigenesis, positioning them as promising candidates for minimally invasive biomarker development across a broad spectrum of dermatological diseases. This narrative review summarises their biological functions and potential clinical applications. The available evidence provides a conceptual basis for an emerging cutaneous “surface liquid biopsy” framework, whilst highlighting the need for standardised sampling, analytical validation, and clinical evaluation before such approaches can be translated into clinical practice. Although ample disease-associated ncRNA signatures have been uncovered, many remain at exploratory or early translational stages, and the concordance of these ncRNA signatures with corresponding blood and tissue biomarkers needs to be established. Continued advances in sampling strategies, analytical technologies, and clinical validation are needed to translate these findings into clinically deployable platforms for dermatological diseases and, potentially, systemic diseases in the future.

## 1. Introduction

The development of minimally invasive diagnostic strategies has become an increasingly prominent focus in biomedical research and clinical practice. Laboratory testing underpins most modern clinical decision-making, and liquid biopsy approaches have expanded molecular profiling by enabling analysis of blood and other biofluids and reducing the need for frequent surgical tissue sampling [1,2]. Whilst blood remains an established matrix for circulating systemic biomarkers, skin-derived biofluids and surface materials are being investigated as potential sources for non-invasive biomarker discovery [3,4].

As the body’s largest organ, the skin forms a highly accessible and dynamic interface between internal physiology and the external environment (Figure 1). It continuously exchanges molecular material through sweat, sebum, desquamating cells, and other surface secretions. Collectively, these materials comprise a complex mixture of proteins, lipids, metabolites, nucleic acids, ions, and other biomolecules that may reflect processes within the skin and, in certain contexts, may also be associated with activity in extradermal or systemic compartments [5]. Various minimally invasive sampling techniques, including tape stripping, skin swabs, suction blister collection, microneedle arrays, and sweat collection patches, have been developed and enable the acquisition of material from the stratum corneum, epidermis, and eccrine sweat glands without requiring punch or excisional biopsies [6]. These approaches can also facilitate repeated sampling of cutaneous sites, creating opportunities for longitudinal monitoring of local molecular changes and treatment responses.

Recent studies suggest that multiple classes of nucleic acids, including microRNAs (miRNAs), long non-coding RNAs (lncRNAs), messenger RNA (mRNA) and cell-free DNA (cfDNA) fragments, can be detected in sweat and skin-associated extracellular matrices, including sweat and interstitial fluid, which are often enriched in extracellular vesicle (EV) fractions, as observed in other biological matrices [7,8,9,10,11]. Encapsulation within EVs or association with ribonucleoprotein complexes may protect these nucleic acids from enzymatic degradation and external stressors (e.g., UV light and chemical insults), enabling their persistence outside the cell. Given their known involvement in many fundamental cellular pathways, including inflammation, immune modulation, tissue remodelling, and tumourigenesis [12,13,14,15,16], detection of these extracellular nucleic acids in accessible skin-associated matrices may provide clinically informative molecular indicators of pathological onset and progression, whilst potentially complementing other biomarker classes.

Other non-invasive diagnostic avenues, such as breath-based profiling, have gained attention for capturing aerosolised nucleic acids and non-coding RNAs within EVs, with specific signatures indicating the presence of cancers, infectious diseases, and metabolic disorders [17]. However, this approach faces recognised technical challenges, including ambient dilution, low analyte abundance, contamination risk, and lack of standardised workflows for collection and nucleic acid discovery [17,18]. In contrast, skin-derived extracellular non-coding RNAs may offer several potential advantages as a non-invasive diagnostic avenue, including the potential for more localised disease-associated signals and direct access to nucleic-acid-containing material at the site of cutaneous pathology [16,19,20]. Dermal interstitial fluid (ISF) is especially attractive because it contains a broad range of biomarkers also found in blood, including overlapping RNA species, although their relative abundance may differ between matrices. Moreover, it can now be accessed by minimally invasive microneedle-based systems compatible with multiplexed or on-patch analysis [6].

This narrative review summarises the current evidence supporting skin-associated extracellular nucleic acids, particularly non-coding RNAs (ncRNAs), as candidates for future biomarker development across tissue and accessible sampling matrices. In addition to outlining the biological functions of skin ncRNAs and the conceptual and methodological landscape of skin-associated sampling, we evaluate nucleic acid signatures associated with dermatological conditions alongside present findings that skin-derived extracellular nucleic acid profiles may also reflect systemic disease processes. Importantly, the review considers evidence across distinct stages of biomarker development, from tissue-based biological and mechanistic associations to extracellular detection and emerging clinical applications. The discussion focuses on peer-reviewed experimental and clinical studies that characterise extracellular nucleic acids in skin-associated matrices whilst considering the emerging concept of the skin as an accessible source for “surface liquid biopsy”.

## 2. Molecular Classes and Roles of Skin Non-Coding RNAs

### 2.1. Extracellular Nucleic Acids in Skin and Sweat

Skin surface secretions and eccrine sweat represent readily accessible outputs that may reflect molecular processes within epidermal and dermal compartments and, potentially, systemic influences. These biofluids arise from coordinated contributions by epidermal and adnexal cells, resident and infiltrating immune cells, commensal microbiota, and ISF within the cutaneous microenvironment [21,22]. As such, they constitute complex biological matrices containing molecules derived not only from superficial tissue layers but also from deeper dermal and epidermal regions, with systemic contributions also possible. These molecules include proteins, lipids, ions, and small metabolites, as well as extracellular nucleic acids of human and microbial origin. The extracellular space, skin surface, and sweat harbour a diverse repertoire of cell-free nucleic acids (cfNAs) that may reflect local skin physiology and, potentially, systemic processes [23]. Whilst intracellular nucleic acids are well-characterised, recent advances in minimally invasive sampling and high-throughput sequencing have unveiled a complex landscape of extracellular DNA and RNA in skin-associated compartments [23]. The detection of these biomolecules in sweat and skin surface secretions demonstrates the feasibility of recovering nucleic acid signals from accessible cutaneous matrices, although their biological origin and clinical relevance may vary according to the sampling compartment. Moreover, approaches such as microneedle-based and microperfusion techniques have demonstrated that ISF contains cell-free nucleic acids that are qualitatively comparable to those detected in blood, supporting the potential for minimally invasive access to tissue-associated molecular information [5,24,25]. Hydrogel-based, porous, and hollow microneedles can repeatedly sample ISF without surgical biopsy, providing a potential route for longitudinal molecular monitoring [10,26,27,28,29]. These findings support the idea that ISF can serve as an accessible intermediary that may reflect both local tissue activity and systemic molecular signatures [10,26,27,28,29,30,31].

Cumulatively, these observations provide a possible conceptual basis for a dynamic skin–ISF–sweat–circulation axis, in which interactions between cutaneous compartments and the systemic circulation may contribute to the chemically rich mixture of nucleic acids, proteins, and metabolites within skin-associated biofluids [22,32]. This molecular continuum provides a framework for investigating skin-associated matrices as accessible sources of disease-associated biomarkers, although the extent to which individual molecular signals reflect local versus systemic processes remains to be established. Within this molecular milieu, extracellular nucleic acids represent a key non-volatile biomolecule class that is becoming increasingly identifiable in surface extracts and eccrine sweat. Early profiling studies have revealed that these matrices harbour diverse nucleic acid species, including non-coding RNAs, particularly miRNAs and lncRNAs, mRNAs, genomic and mitochondrial DNA (mtDNA) fragments, and microbial nucleic acids derived from the resident microbiota. cfDNA and mtDNA released into adjacent cutaneous fluids are thought to arise from routine cellular turnover, inflammation-mediated cell death, oxidative stress, and overt tissue injury in the epidermis and dermis, whilst circulating sources may also contribute to extracellular nucleic acid pools within cutaneous compartments [33,34]. However, the extent to which nucleic acids detected in skin-associated fluids originate locally or reflect systemic circulation remains to be established. As in plasma and other biofluids, extracellular nucleic acids in skin-associated matrices may be protected through association with ribonucleoprotein complexes and/or encapsulation within EVs, which can reduce degradation by nucleases and environmental stressors [21,33,34].

Circular RNAs (circRNAs), which are generated through back-splicing events that produce covalently closed RNA loops, represent another notable class of regulatory RNAs. These molecules can function as miRNA sponges, interact with RNA-binding proteins, or in some cases serve as templates for limited translation [35,36,37]. Additional ncRNA species—including small nucleolar RNAs (snoRNAs), small nuclear RNAs (snRNAs), and PIWI-interacting RNAs (piRNAs)—participate in RNA processing, modification, and genome defense, although their roles in cutaneous biology remain comparatively understudied [32,33,38]. In the skin, these mechanistic categories are exemplified by differentiation-associated lncRNAs such as *ANCR* [39] and *TINCR* [40,41], which maintain keratinocyte progenitor states or stabilise differentiation-associated transcripts through STAU1 binding, as well as by circRNAs such as *ciRS-7/CDR1as* [34], which act as miRNA sponges in inflammatory skin disease.

### 2.2. ncRNA-Mediated Gene Regulation and Intercellular Communication in Skin

Skin-derived non-coding RNAs comprise several major regulatory RNA classes with distinct biogenesis pathways and mechanisms of action. miRNAs are short RNAs (approximately 22 nucleotides (nt)) generated through sequential processing of primary transcripts by the RNase III enzymes Drosha and Dicer, with Drosha acting in the nucleus and Dicer in the cytoplasm. miRNAs typically regulate gene expression by guiding the RNA-induced silencing complex (RISC) to specific mRNAs, though target recognition is mediated through partially complementary base pairing in animal models [42,43]. This interaction silences the target gene by either triggering the degradation of the mRNA or physically blocking it from being translated into protein. miRNAs exert broad regulatory effects within the skin, modulating processes including keratinocyte proliferation and differentiation, oxidative stress responses, inflammatory signalling, angiogenesis and apoptosis. Dysregulated miRNA expression has been documented across a wide range of dermatologic conditions [33]. In mesenchymal stromal cells and related lineages, miRNAs influence stemness, lineage commitment, and paracrine signalling, thereby shaping tissue repair and inflammatory responses within the skin [22].

lncRNAs, defined as non-protein-coding transcripts longer than 200 nt, are typically transcribed by RNA polymerase II and often undergo splicing and polyadenylation. They regulate gene expression through multiple mechanisms, including acting as scaffolds for chromatin-modifying complexes, guiding transcriptional regulators to specific genomic loci, sequestering transcription factors or RNA-binding proteins, and functioning as competing endogenous RNAs that modulate miRNA activity [24,35,44,45,46]. Large-scale RNA sequencing (RNA-seq) analyses of normal, uninvolved, and psoriatic lesional skin detected approximately 4000 skin-expressed lncRNAs, including over 1000 previously unannotated species, many of which exhibited strong epidermal and immune-associated co-expression signatures and localisation to the epidermal differentiation complex [22,47]. In psoriasis keratinocytes, RNA-seq found 889 differentially expressed lncRNAs compared with healthy keratinocytes. Functional validation uncovered that the IL-17A–inducible lncRNA *CYDAER* (RP11-295G20.2) is enriched in suprabasal layers and is dynamically regulated during differentiation, whilst its knockdown promotes terminal differentiation, directly implicating lncRNAs in epidermal maturation [21].

A distinctive feature of ncRNA biology in skin is the involvement of ncRNAs in intercellular communication mediated by EVs. Keratinocytes, fibroblasts, melanocytes, immune cells and tumour cells release EVs containing miRNAs, lncRNAs and circRNAs, enabling transfer of regulatory molecules across the local microenvironment [22,24,33,34]. EV-associated ncRNAs may therefore provide a mechanistic link between intracellular regulatory processes and extracellular molecular signatures. Circulating EV-associated miRNAs also mediate intercellular communication in both local and systemic contexts, and their detection in accessible biofluids has prompted investigation of their potential as liquid biopsy biomarkers [5,9,25,47,48,49,50,51]. In melanoma and other skin cancers, EV-associated ncRNAs mediate tumour–stroma cross-talk and contribute to angiogenesis, immune modulation and pre-metastatic niche formation, with distinct EV ncRNA signatures reported as candidate diagnostic or prognostic biomarkers [24,44]. Emerging evidence also indicates that EV-mediated RNA transfer contributes to physiological processes such as wound repair and skin ageing. Senescent dermal fibroblasts release EVs enriched in miRNAs such as *miR-23a-3p*; these EVs can be internalised by keratinocytes and modulate pathways involved in wound closure and epidermal differentiation [33].

Collectively, these findings support a model in which ncRNAs regulate gene expression within cells, mediate intercellular communication via EVs, and contribute to extracellular pools of nucleic acids detectable in skin-adjacent fluids such as sweat and surface secretions [33,34,44]. Targeted studies have detected representative ncRNA species spanning skin tissue and sweat, providing evidence that ncRNAs can be recovered from both tissue and accessible extracellular matrices, although these findings represent distinct levels of evidence and do not by themselves establish clinical biomarker validity. Their tissue specificity, responsiveness to inflammatory and oncogenic cues, and potential stabilisation within vesicular carriers make them attractive candidates for biomarker development for dermatological diseases [24,25,34,45,48,52]. At the same time, the complexity of ncRNA regulatory networks and challenges in their detection and standardisation emphasise the need for careful validation and methodological harmonisation [34,35,36]. Cumulatively, these insights provide a conceptual foundation for the subsequent sections examining the biomarker potential and translational development of skin-derived nucleic acids.

A PubMed search of English-language articles published between 2015 and 2025 was used to identify relevant literature informing this review. A total of 696 publications were identified, of which 183 were retained following screening. Additional landmark publications outside this period were included where they provided important foundational or mechanistic evidence. The literature identified was predominantly focused on dermatological conditions, including melanoma, psoriasis, inflammatory skin diseases, and nonmelanoma skin cancers, whilst a smaller body of work has examined other cutaneous disorders and physiological contexts. The subsequent sections therefore synthesise representative studies according to the biological functions, biomarker applications, and translational potential of skin- and sweat-derived nucleic acids. miRNAs remain by far the most extensively investigated class of extracellular nucleic acids, with growing evidence on the diagnostic and mechanistic relevance of lncRNAs and circRNAs. In contrast, relatively little attention has been devoted to other extracellular nucleic acid classes, including cell-free DNA (cfDNA), mtDNA, and less well-characterised regulatory RNAs.

Collectively, the available literature indicates that although skin-derived nucleic acid biomarker research has expanded considerably, the evidence base remains concentrated in tissue-based and mechanistic studies, whilst comparatively fewer investigations have examined minimally invasive sampling matrices such as sweat and dermal ISF. The following sections integrate representative studies to examine the biological functions, diagnostic performance, and translational potential of skin- and sweat-derived nucleic acid biomarkers.

### 2.3. Skin- and Sweat-Derived miRNAs as Disease Biomarkers

Amongst skin- and sweat-derived nucleic acids, miRNAs, which post-transcriptionally regulate gene expression and are consistently dysregulated across a wide range of conditions, are promising candidates for biomarker development [53,54,55]. Their small size, relative extracellular stability, tissue- and disease-specific expression patterns, amenability to sensitive molecular detection, and involvement in fundamental cellular pathways—including inflammation, immune modulation, tissue remodelling, and cancer progression—make them particularly attractive molecular indicators of pathological changes [48,49,55,56]. Current research mainly focuses on two complementary sources of miRNA biomarkers: skin-associated tissues and accessible extracellular matrices, including skin surface secretions and sweat. Accumulating evidence suggests that miRNAs detected in skin surface secretions and sweat may capture signals from cutaneous and immune cells, offering a minimally invasive window into tissue dynamics and disease-associated processes. Table 1 lists selected studies demonstrating the potential for and progress towards clinical translation (i.e., diagnostic, prognostic, and treatment-monitoring applications) of skin- and sweat-derived miRNAs, including patient-based investigations, clinical validation, diagnostic performance, and experimental evidence.

Skin-derived miRNAs, sampled from skin biopsies, tape stripping or ISF, have been extensively investigated in a variety of inflammatory and malignant dermatological diseases, systemic diseases, and in forensics. Sonkoly et al. [58] revealed *miR-203* as a specifically skin-enriched miRNA that is markedly upregulated in psoriatic lesions, marking it as a strong biomarker and therapeutic target candidate. Subsequent work by the same group found that *miR-155* is highly overexpressed in atopic dermatitis lesions, underscoring its association with chronic cutaneous inflammation [59]. Utilising deep sequencing of human skin biopsies to map the entire “miRNAome” of healthy vs. psoriatic skin, Joyce et al. [61] identified *miR-31* and *miR-21* as strong candidate biomarkers for the disease. Li et al. [38] demonstrated that miR-132 promotes the transition from inflammation to proliferation during skin wound healing, supporting a potential role for this miRNA in wound-repair regulation. More recently, Chicharro et al. [65] revealed that tissue *miR-135b-5p* expression decreases following biologic therapy and correlates with clinical improvement, reinforcing its potential as a treatment-response biomarker.

Beyond inflammatory dermatoses, skin-derived miRNAs have also emerged as valuable diagnostic and prognostic biomarkers in cutaneous malignancies, particularly melanoma and squamous cell carcinoma [67,69]. Collecting upper-layer skin cells from pigmented lesions using adhesive tape stripping (instead of biopsies), Wachsman et al. [69] found that elevated *miR-211-5p* and *miR-155* levels could accurately distinguish melanoma from benign moles. Evaluating primary melanoma, Dika et al. [70] demonstrated that combined tissue expression of *miR-21-5p* and *miR-146a-5p* correlated with Breslow thickness and predicted overall survival in patients with superficially spreading melanoma. Similarly, Hanna et al. [63] noted that reduced *miR-205* expression independently predicted poorer melanoma-specific survival, reinforcing its role as a prognostic tissue biomarker.

Dermal ISF, the extracellular fluid occupying the interstitial space between skin cells, can now be sampled using microneedle patches and microperfusion technologies [10,71,72,73,74]. Hydrogel-coated microneedle patches functionalised with sequence-specific peptide nucleic acid probes can achieve rapid ISF uptake (approx. 0.97–6.5 μL within minutes) and on- or off-patch quantification of cfNAs and miRNAs at nanomolar levels [10,71]. Related ‘lab-on-a-microneedle’ architectures integrating paper microfluidics or electrochemical strips already facilitate on-chip detection of canonical ISF biomarkers (e.g., glucose, lactate, and pH), underscoring the feasibility of extending these designs to direct nucleic-acid measurement in vivo [72,73]. Qiao et al. [57] developed hydrogel microneedles to painlessly extract skin ISF and successfully detected three types of psoriasis-related miRNAs either directly on the patch or in solution after release from the hydrogel using a catalytic hairpin assembly signal amplification reaction. Using a microneedle sensor array, Li et al. [52] efficiently measured ISF miRNA and Cu^2+^ levels in agarose skin and fresh porcine cadaver skin models. Collectively, these studies underline the feasibility of analysing ncRNAs within skin ISF, supporting its potential as a minimally invasive sampling matrix for future dermatological biomarker development.

Beyond direct ncRNA detection, EVs isolated from skin and dermal interstitial fluid have emerged as important mediators of intercellular communication. Terlecki-Zaniewicz et al. [12] documented the successful isolation of EVs from human skin tissue and dermal ISF and showed that fibroblast-derived EV miRNAs are transferred to keratinocytes both in vitro and in three-dimensional human skin equivalents. Furthermore, EVs released by senescent fibroblasts modulated keratinocyte behaviour, including wound closure and epidermal differentiation, highlighting an important role for EV-mediated signalling within the cutaneous microenvironment. Although this was primarily a mechanistic study, the findings establish the feasibility of isolating EVs from skin-associated matrices and encourage their future investigation as carriers of clinically relevant dermatological biomarkers.

As two of the most consistently dysregulated EV-associated miRNAs in psoriasis, *miR-146a* and *miR-31* play critical roles in mediating cross-talk between skin cells and the immune system in psoriasis [75]. From wound exudate fluid collected directly from the skin of diabetic foot ulcers, Liu et al. [60] isolated EVs containing dysregulated *miR-195-5p* and *miR-205-5p*, which impaired angiogenesis and were associated with delayed wound healing.

Sweat represents another emerging, minimally invasive biofluid for molecular diagnostics. Although there is less research on cfNA in sweat compared to blood due to the very low nucleic acid concentrations, high RNase/DNase activity on the skin, and the difficulty of collecting large volumes of pristine sweat, a landmark study by Weber et al. [76] first observed the presence of stable, detectable miRNAs in human sweat and established a baseline sweat miRNA profile. Further studies have shown that sweat miRNAs can be associated with EVs, which may help protect them from degradation by RNases. Targeted analyses have confirmed that canonical endothelial- and immune miRNAs—including *miR-21-5p*, *miR-26a-5p*, *miR-126-3p*, *miR-146a-5p*, *miR-221-3p and miR-222-3p*—are exported in EVs into sweat, with sweat EV-associated *miR-21-5p* and *miR-26a-5p* increasing in response to acute endurance exercise stimuli of different intensities [8]. These observations indicate that sweat-borne miRNAs, mainly EV-associated rather than HDL-bound, can track rapid physiological perturbations such as exercise-induced stress and vascular activation. Similarly, Wu et al. [77] detected exosomes packed with *miR-214* and *miR-21* in human sweat, which were both implicated in local immune regulation and skin inflammation. Building upon these results, several groups subsequently developed wearable biosensors for real-time sweat miRNA detection. For example, Zhao et al. [78] developed a flexible photoelectrochemical biosensor for ultrasensitive miRNA (miR-21) detection, with a lower detection limit of 0.41 fM, illustrating the translational potential of sweat-based miRNA sensing for future point-of-care diagnostics.

### 2.4. Skin-Derived lncRNAs Serve as Disease Biomarkers

Large-scale RNA sequencing studies of human skin biopsies have characterised numerous lncRNAs that are expressed within skin tissue and associated extracellular materials [45,79,80]. Analysis of normal and psoriatic skin lncRNAs by Tsoi et al. [45] uncovered distinct tissue-specific expression patterns and epigenetic signatures, underscoring the regulatory complexity of the cutaneous lncRNA landscape. Similarly, by examining the epidermis of patients with psoriasis and healthy donors, two groups independently identified *PRINS* (Psoriasis susceptibility-related RNA Gene Induced by Stress), one of the first lncRNAs explicitly related to a skin disease, and provided evidence that PRINS plays a role in the keratinocyte stress response and psoriasis pathogenesis [81,82]. Leucci et al. showed that *SAMMSON* is consistently co-gained with melanoma-specific oncogene *MITF*, and promotes tumour progression through interaction with *p32*, a master regulator of mitochondrial homeostasis and metabolism [83]. Table 2 summarises selected studies illustrating the growing clinical potential of skin-derived lncRNAs as disease biomarkers. To date, the strongest translational evidence has emerged from melanoma diagnostics. Several research groups have reported the clinical feasibility of using *LINC00518* as part of an adhesive patch assay that non-invasively collects cells from a mole for melanoma diagnosis [84,85]. More recently, Wu et al. [86] reported a seven-hypoxia-associated-lncRNA signature comprising lncRNAs *AC008687.3*, *AC009495.1*, *AC245128.3*, *AL512363.1*, *LINC00518*, *LINC02416*, and *MCCC1-AS1* that stratified melanoma patients according to prognosis. Through genome-wide analyses, Wang et al. [87] characterised the biological function of *LINC00518* in melanoma. Liu et al. [88] observed that *LINC00518* also induces radioresistance in melanoma by regulating glycolysis through an *miR-33a-3p/HIF-1α* negative feedback loop. Beyond its biological role, LINC00518 has also been incorporated into a commercially available clinical assay, the DermTech Pigmented Lesion Assay, which uses an adhesive patch to evaluate suspicious pigmented lesions [84,85,89]. In addition, work by Flockhart et al. [90] identified *BANCR* (BRAF-activated non-protein-coding RNA) as another highly overexpressed biomarker in melanoma tissue.

Beyond melanoma, lncRNAs have been implicated in cutaneous squamous cell carcinoma, diabetic wound repair, and fibrotic skin disease, although most evidence remains mechanistic rather than clinically validated. Chen et al. [91] found that *HOTAIR* is upregulated in tumour and recurrent tissue, correlates with poorer survival, and promotes cancer stemness and progression through the Sp1/DNMT1/miR-199a-5p pathway. A similar role for the *THOC7-AS1/OCT1/FSTL1* axis in promoting EMT was reported by Yu et al. [92], suggesting that it has potential as a therapeutic target in squamous cell carcinoma.

In diabetic wound healing, evidence is at an earlier stage. lncRNA *XIST*, which was downregulated in diabetic ulcer rat skin tissues and high-glucose-induced HaCaT cells, promotes cell proliferation and migration through the *miR-126-3p/EGFR* axis, suggesting a role in delayed repair rather than establishing a validated clinical biomarker [93]. Similarly, keratinocyte-derived exosomal *MALAT1* was reported to enhance diabetic wound healing through upregulation of MFGE8, highlighting its biological relevance in tissue repair [94].

In fibrotic skin disease, *TSIX* is overexpressed in systemic sclerosis dermal fibroblasts and appears to promote collagen accumulation by stabilising type I collagen mRNA, supporting its relevance as a candidate fibrosis-associated biomarker and mechanistic regulator [95]. Consistent with this finding, Messemaker et al. [96] also identified increased *TSIX* expression in skin fibroblasts from patients with systemic sclerosis, further supporting its association with fibroblast activation and skin fibrosis.

### 2.5. Potential Role of Other cfNAs in Skin and Sweat as Disease Biomarkers

Although most studies have focused on miRNAs and lncRNAs, these species coexist with a broader repertoire of extracellular nucleic acids, including cfDNA and mtDNA, along with co-secreted proteins and metabolites that jointly encode information about barrier integrity, immune activation, microbial interactions, and nascent tumourigenic changes in the cutaneous microenvironment [21,22,33,34]. Consequently, there is growing interest in exploiting other extracellular nucleic acids as complementary biomarkers of disease.

Recent studies have explored the diagnostic potential of circulating tumour DNA (ctDNA) or cfDNA in skin-associated biofluids, particularly dermal ISF [97,98]. Through in situ microneedle sampling of dermal ISF, Wu et al. [97] demonstrated the potential of ISF ctDNA as a biomarker for tumour diagnosis. Employing a similar sampling strategy using wearable microneedle patches, Yang et al. [98] detected Epstein-Barr virus cell-free DNA (EBV cfDNA) in ISF, illustrating the broader applicability of ISF-based nucleic acid detection for both oncological and infectious diseases. Although investigation of cfDNA in skin-associated biofluids remains at an early stage, these studies point to the potential of extracellular nucleic acids beyond ncRNAs. In practice, integrating miRNA and lncRNA signatures with other nucleic acid and protein markers from the same sample is likely to yield more specific and mechanistically interpretable biomarker panels than any single analyte class alone and provide a conceptual bridge to multi-omic, skin-based liquid biopsy strategies.

## 3. Challenges and Limitations

Currently, the research on skin- or sweat-derived nucleic acids is still in its infancy stage, with limited evidence supporting immediate clinical application despite encouraging advances [5,51]. At present, many studies still use synthetic or spiked oligonucleotides, rather than genuine disease-derived signatures, as standards for ncRNA quantification. Furthermore, many analytical platforms have been primarily evaluated in animal models, ex vivo skin, or small pilot studies, with a lack of disease-specific validation and head-to-head comparisons with established liquid biopsy assays.

First, the concentration of cell-free nucleic acids in skin and sweat is markedly lower than in blood plasma or serum. Whilst blood may contain picomolar to nanomolar concentrations of specific miRNAs, sweat often contains trace amounts at femtomolar (10^−15^) or even attomolar (10^−18^) levels. Detecting such minute quantities often requires substantial signal amplification, which may increase background noise and the risk of false-positive results. Due to these ultra-low miRNA levels in sweat, ISF has emerged as an attractive alternative sampling matrix. However, extracting ISF requires microneedle arrays. Whilst minimally invasive, microneedle-based sampling typically yields relatively small fluid volumes (ranging from nanolitres to a few microlitres), making standard lab-based RNA extraction and sequencing technically demanding without the use of specialised microfluidics. Additionally, sampling the skin surface is analytically challenging due to the abundance of RNases, dense microbial colonisation, the potential for environmental contamination, and complex extracellular matrix, all of which can compromise nucleic acid yield, integrity, and purity [62,68]. Despite these hurdles, effectively incorporating skin and sweat nucleic acid features—such as cfDNA fragments or mtDNA signatures—or miRNA panels into diagnostic frameworks could significantly increase clinical specificity, directly linking surface-accessible readouts to defined pathways in dermatologic, oncologic, and metabolic diseases.

Second, sampling methodology and data normalisation represent important analytical and engineering challenges when monitoring dynamic changes in surface-derived miRNAs and lncRNAs [23,64]. Unlike blood, the volume of which is relatively constant, sweat volume fluctuates dramatically based on environmental temperature, emotional state, hydration status, and physical exertion. Furthermore, as sweat emerges onto the skin, water evaporates, artificially concentrating the biomarkers left behind. This dynamic makes calculating absolute miRNA concentrations highly complex. Moreover, analytical results are frequently compromised by biological contamination. The skin surface is coated in sebum (lipids), dead stratum corneum cells, and a dense cutaneous microbiome comprising bacteria and fungi. When harvesting sweat or utilising skin tape-stripping, exogenous bacterial RNA and lipid interference can severely inhibit the downstream biochemical reactions required to accurately detect human miRNAs.

Third, given the unique properties of skin- and sweat-derived samples, sample collection conditions and methods are critical for developing potential disease biomarkers. Matrix-specific variables—including anatomical site, lesional versus non-lesional sampling, sweat induction method, sweat rate, ambient temperature, hydration status, physical activity, topical products, skin cleansing, collection duration, tape-strip depth, microneedle design, blood contamination, storage conditions, freeze–thaw exposure, extraction efficiency, and EV-isolation method—should be carefully considered and monitored.

Another challenge concerns the development of wearable biosensing technologies capable of continuous, real-time nucleic acid monitoring. Although considerable progress has been made in microfluidic and electrochemical sensor design, achieving reliable long-term analytical performance under physiological conditions remains difficult. Sensor surfaces are susceptible to biofouling caused by the accumulation of proteins, lipids, and cellular debris, which progressively reduces analytical sensitivity. Simultaneously, fluctuations in sweat composition—including changes in pH, ionic strength, and salinity—can alter sensor performance and measurement stability.

Taken together, the successful clinical translation of skin- and sweat-derived nucleic acid biomarkers will depend on continued advances in molecular biology, analytical chemistry, microfluidics, biosensor engineering, and computational data analysis. As detection technologies mature and methodological standardisation improves, skin-based liquid biopsy approaches may ultimately complement, rather than replace, existing blood-based diagnostics by enabling less invasive, longitudinal monitoring of both skin and systemic diseases. Continued integration of multi-omic biomarkers with wearable sensing technologies may ultimately establish skin as an accessible platform for precision diagnostics and personalised disease monitoring.

## 4. Conclusions and Perspectives

Accumulating evidence supports the potential of extracellular nucleic acids derived from skin and sweat as a burgeoning class of minimally invasive biomarkers, underpinning the concept of cutaneous “surface liquid biopsies” for dermatological and, potentially, systemic diseases. This narrative review highlights the rapid expansion of this field whilst revealing that a substantial proportion of current research remains focused on tissue-based discovery and mechanistic studies, with comparatively limited emphasis on surface sampling, prospective clinical validation, and implementation-focused research. Together, these findings indicate that although skin-derived nucleic acid biomarker research has matured considerably, translation into clinically deployable diagnostic platforms remains at an early stage.

Data from in vitro systems, animal models, and early human studies indicate that keratinocytes, melanocytes, immune cells, and adnexal structures can contribute to EVs and cell-free nucleic acids under both physiological and pathological conditions. Within this microenvironment, miRNAs and other small RNAs act as central mediators of intercellular communication at the skin barrier. As depicted in Figure 2, extracellular nucleic acid analysis typically involves skin or sweat collection, EV enrichment where applicable, nucleic acid extraction, and downstream sequencing or targeted assays for biomarker discovery and monitoring. However, despite rapid technological progress, translating these diagnostic modalities into clinical practice faces substantial methodological and regulatory barriers. To realise the vision of “surface liquid biopsies,” coordinated progress across clinical cohorts, mechanistic biology, device engineering, and standardisation must be achieved.

### 4.1. Large-Scale Cohort Studies

Currently, most of the evidence in this field is cross-sectional, relying on modest cohort sizes and predominantly correlational outcomes. There is a notable lack of large, longitudinal trials demonstrating clinical-grade diagnostic performance for skin- or sweat-based miRNA or lncRNA assays. Therefore, establishing these nucleic acids as reliable dermal disease biomarkers will require substantial, disease-focused human cohorts and prospective investigations to strictly verify diagnostic cut-offs. Reported diagnostic performance estimates should be interpreted in the context of study design and cohort size. High AUC, sensitivity, or specificity values from retrospective, selected, or small cohorts may overestimate performance and should not be considered evidence of clinical utility without independent or prospective validation. Where reported by the primary studies, confidence intervals have been included to convey uncertainty around these estimates.

### 4.2. Integration with Multi-Modal Biomarker Profiles

Whilst the primary advantage of surface liquid biopsies lies in their minimally invasive nature and ease of collection, their diagnostic performance and clinical value can be significantly enhanced if continuous or repeated sweat and ISF samples are integrated with complementary molecular and physiological data. This includes combining them with blood-based biomarkers, proteomic or metabolic profiles, and parallel physiological data streams, such as heart rate, physical activity, and temperature. This multi-modal information can be analysed using machine learning to infer latent physiological states and derive complex digital biomarkers. Because machine learning-enabled biosensor platforms are already being utilised for stress, cardiometabolic monitoring, and infection surveillance through non-invasive signals, future studies could investigate whether similar architectures can incorporate sweat- or ISF-derived nucleic acid measurements into personalised digital health pipelines. Such approaches would require rigorous assessment of overfitting, calibration, external validation, domain shift, and performance against clinically meaningful comparators before clinical implementation [63,66,67,99,100,101,102].

### 4.3. Potential Biomarkers for Systemic Diseases

Given that circulating EVs have been implicated in inter-organ communication [103,104], it is biologically plausible that ncRNAs associated with distant dysfunctional organs could ultimately be detectable in skin-associated biofluids; however, direct transport from a distant organ to sweat or ISF remains to be established. Consequently, skin- and sweat-derived nucleic acids could potentially be used beyond dermatological disorders, including for systemic disease monitoring. Although direct evidence for systemic disease applications is still developing, preclinical and early human studies—alongside in vitro and ex vivo characterisations of skin-derived EVs—support further investigation of sweat and ISF nucleic acids as potential adjuncts to conventional liquid biopsies. Future research should explicitly determine the extent to which nucleic acid signatures mirror circulating biomarkers, distinguishing true molecular concordance from associations arising through anatomical proximity or vascular exchange. Ultimately, well-phenotyped, longitudinal studies integrating skin and sweat analyses with blood- and tissue-based biomarkers are essential to clarify exactly when skin-derived nucleic acids reflect systemic disease activity versus localised cutaneous pathology.

## Figures and Tables

**Figure 1:**
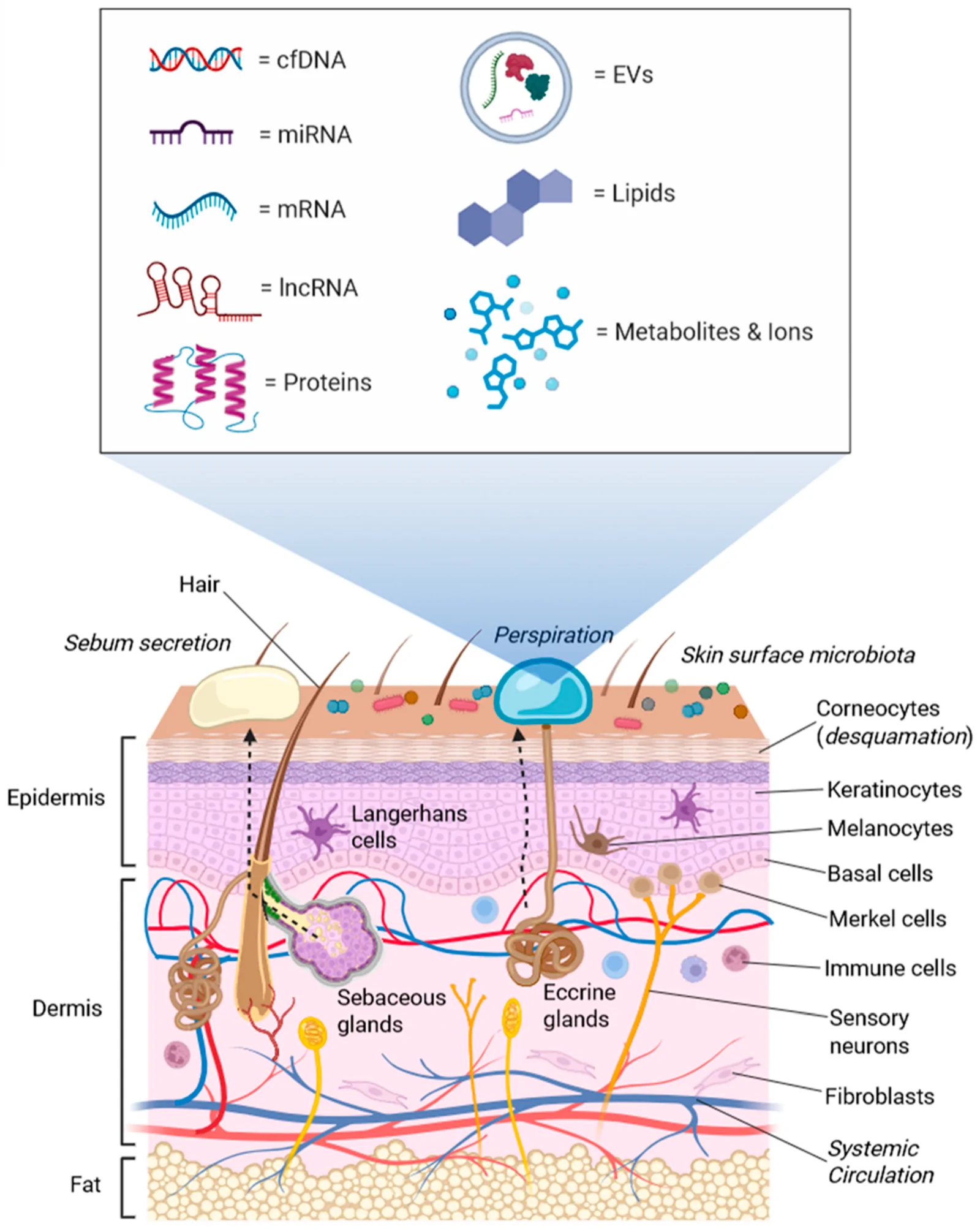
Skin surface secretions integrate cutaneous and potentially systemic inputs. The skin is a dynamic interface that continuously releases biomolecules through sweat, sebum and desquamation. These secretions arise from multiple sources, including keratinocytes, immune cells, melanocytes, the skin microbiota, and potentially biomolecules reaching cutaneous compartments from the systemic circulation. Transported through eccrine and sebaceous glands or shed as corneocytes, these materials accumulate on the surface to form a biofluid matrix composed of nucleic acids, proteins, lipids, metabolites, and ions. This composition allows the skin surface to serve as a non-invasive platform for molecular profiling and biomarker discovery. The depiction of systemic inputs represents a conceptual model rather than established transport of specific nucleic acids from distant organs.

**Figure 2:**
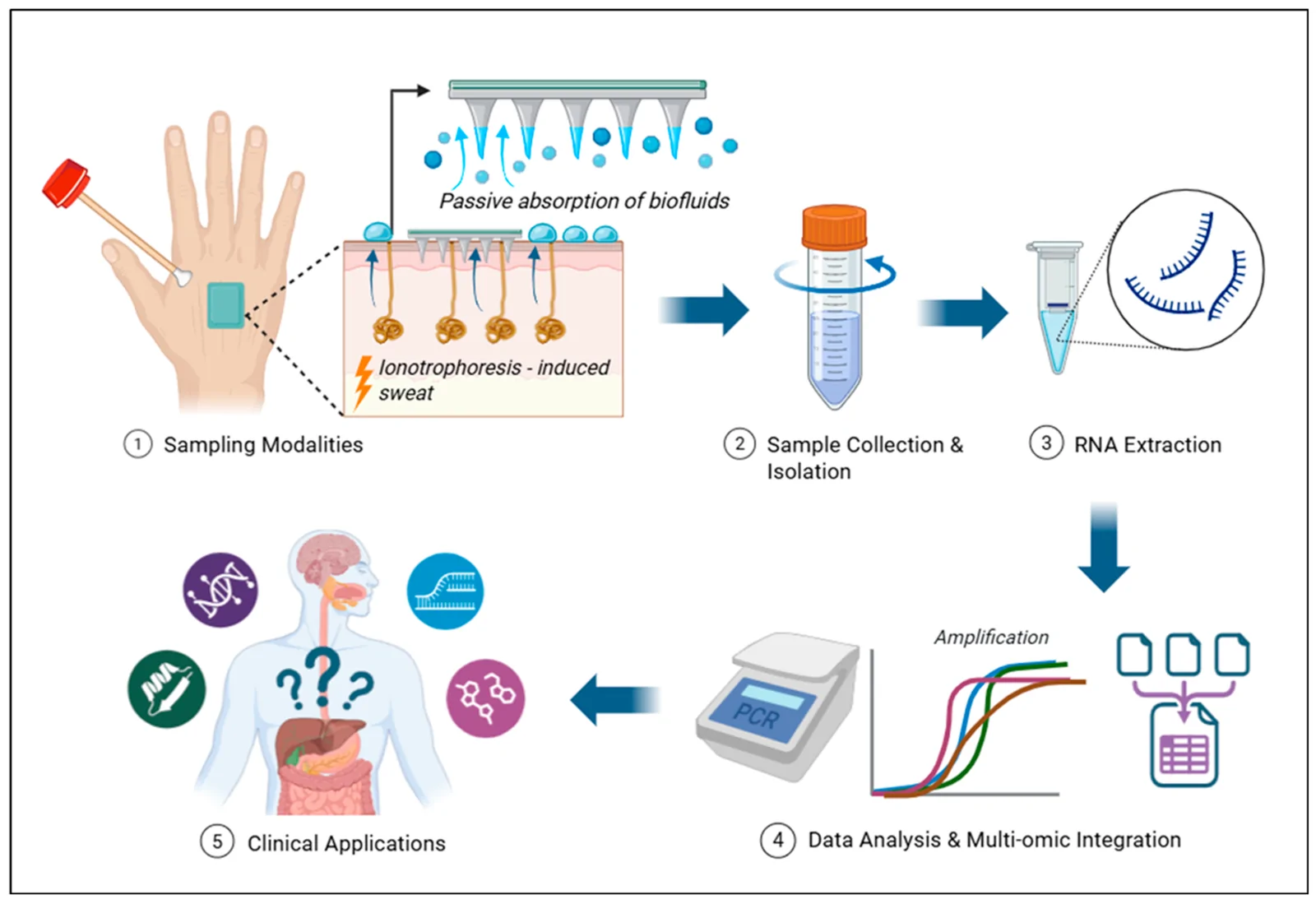
Integrated pipeline for analysing skin surface secretions and sweat-derived nucleic acids for minimally invasive diagnostics. Skin surface secretions and sweat can be collected using minimally invasive approaches such as tape stripping, swabs, sweat patches, and microneedle-based ISF sampling. Collected material undergoes pre-analytical processing to enrich EVs and other protective carriers that stabilise extracellular nucleic acids in nuclease- and microbe-rich environments. Downstream extraction and sequencing or targeted assays (e.g., qPCR and small RNA-seq) enable profiling of RNAs and other nucleic acids present in the sample. Resulting signatures can be interpreted alongside co-measured metabolites and proteins to capture information on both local cutaneous physiology and selected systemic processes, forming the basis of a “surface liquid biopsy” framework. This framework will require the development of standardised sampling and normalisation protocols, and analytical workflows before clinical translation.

**Table 1:** Skin- and sweat-derived miRNA biomarkers with diagnostic, prognostic, or therapeutic relevance.

Authors (Year)	Sample	Biomarker(s)	Disease/Activity	Clinically/Diagnostically Relevant Findings	Intended Use; Evidence Level	Cohort Size	Ref.
Qiao et al. (2022)	ISF	*miR-21*, *miR-141*, *miR-155* (*miR-181* used as an internal control)	Psoriasis	Hydrogel microneedle patch for minimally invasive sampling and in situ fluorescence detection	Technical proof-of-concept; preclinical animal study	Imiquimod-induced psoriasis mice vs. petrolatum controls (n = 3)	[57]
Sonkoly et al. (2007)	Skin	*miR-203*, *miR-146a*, *miR-21*, *miR-125b*	Psoriasis	Psoriasis lesions have a distinct miRNA signature; keratinocyte *miR-203* is linked to reduced *SOCS-3* levels	Biomarker discovery; experimental validation (qPCR)	Discovery microarray: 3 psoriasis patients, 3 eczema patients, 4 controls; validation qPCR: 25 psoriasis patients, 20 eczema patients, 26 controls	[58]
Sonkoly et al. (2010)	Skin	*miR-155*	Atopic dermatitis (AD)	*miR-155* is an AD-associated skin biomarker and pathogenic regulator (targets CTLA4 and promotes T cell proliferation)	Biomarker discovery/mechanistic; experimental validation	18 AD patients, 29 healthy individuals	[59]
Li et al. (2015)	Skin	*miR-132*	Skin wound healing	*miR-132* was upregulated during wound healing and promoted the transition from inflammation to proliferation	Biomarker discovery/mechanistic; Human and preclinical experimental evidence	Human cohort: 14 volunteers (healthy); mouse model: miR-132 knockout (18) vs. wild-type (11)	[38]
Liu et al. (2021)	Skin EVs	*miR-195-5p*, *miR-205-5p*	Diabetic foot ulcers	These miRNAs inhibit VEGFA, angiogenesis, and wound healing in diabetic foot ulcers	Mechanistic/therapeutic; preclinical experimental evidence	Serum/wound fluid from DF/DM patients and controls; cutaneous wounds evaluated in male rats treated with EVs	[60]
Joyce et al. (2011)	Skin	*miR-21*, *miR-31*, *miR-142-3p*, *miR-223/223* *, *miR-378*, *miR-100*, *miR-135b*, *miR-203*, *miR-203-AS*	Psoriasis	Dysregulated miRNAs link to inflammation, epidermal differentiation, and angiogenesis	Biomarker discovery; tissue-based profiling	67 skin biopsies: 20 normal, 23 uninvolved psoriatic, 24 involved psoriatic	[61]
Kozubek et al. (2013)	Skin	*miR-211-5p*, *miR-203*, *miR-205-5p*, *miR-204-5p*, *miR-23b-3p*, *miR-26a-5p*, *miR-26b-5p*, *miR-451a*, *miR-144-3p*, *miR-181b-5p*, *miR-320a/c/d*	Cutaneous melanoma	*miR-211-5p* distinguished primary melanoma (AUC = 0.933), melanoma (AUC = 0.982), and dysplastic nevi (AUC = 0.951) from common nevi, respectively; a 40-miRNA signature classified benign versus malignant lesions and primary versus metastatic melanoma.	Diagnostic; discovery and internal validation	Discovery cohort: 28 samples; validation cohort: 101 skin specimens comprising 19 normal skin, 16 common nevi, 19 dysplastic nevi, 17 melanoma, and 30 primary melanoma samples	[62]
Hanna et al. (2012)	Skin	*miR-205*	Cutaneous melanoma	Low *miR-205* expression associated with poorer survival	Prognostic; discovery and validation cohort	Discovery cohort: 105 melanoma patients; validation cohort: 206 melanoma patients	[63]
Love et al. (2024)	Skin	*MEL38*, *MEL12*	Cutaneous melanoma	*MEL38* distinguished invasive melanoma from benign lesions and melanoma (AUC = 1.00 in tissue and 0.99 in plasma); *MEL12* outperformed some previously published prognostic models	Diagnostic/prognostic; discovery and independent external validation	Discovery/validation cohort: 124 samples (60 FFPE tissue, 64 plasma); external validation: 548 tissue samples and 217 plasma samples	[64]
Chicharro et al. (2020)	Skin	*miR-135b-5p*	Plaque psoriasis	Decreased *miR-135b-5p* levels associated with PASI improvement, independently predicting clinical response to treatment	Treatment-response/prognostic; discovery, validation, and longitudinal treatment-response assessment	Discovery: 5 psoriasis patients, 5 controls; validation: 44 patients; treatment response: 15 patients with repeat biopsies	[65]
Liu et al. (2022)	Skin, blood	*miR-203*	Diabetic foot ulcers	*miR-203* upregulated in foot wounds, suggesting that *miR-203* may serve as biomarkers for monitoring treatment response and predicting outcomes	Treatment-response/prognostic; clinical observation and treatment-response assessment	96 patients with diabetic foot ulcers, 76 patients without foot ulcers, 52 non-diabetic patients with chronic skin ulcers, and 50 controls	[66]
Pinto et al. (2015)	Skin	*miR-101*, *miR-221*, *miR-21*, *miR-338-3p*, and *miR-191*	Melanoma	Identified predictive biomarkers to stratify melanoma patients at higher risk of shorter response duration in anti-BRAF therapy	Predictive/treatment-response; biomarker discovery and treatment-response assessment	30 BRAF-mutated and 13 BRAF wild-type; 20 BRAF-mutated patients treated with vemurafenib	[67]
Lago et al. (2018)	Skin	*miR-361-3p*	Cutaneous leishmaniasis	*miR-361-3p* associated with poor healing (AUC = 0.95, 81.2% sensitivity [95% CI: 54.3–95.9], 100% specificity [95% CI: 39.7–100])	Prognostic/treatment-response; clinical observational evidence	24 patients (20 treatment responders, 4 failures)	[68]
Karvinen et al. (2020)	Sweat EVs	*miR-21-5p*, *miR-26a-5p*, *miR-126-3p*, *miR-146a-5p*, *miR-221-3p*, *miR-222-3p*	Endurance exercise	*miR-21* and *miR-26* levels changed with endurance exercise intensity	Biomarker discovery; early human/pilot study	8 participants (3 men and 5 women)	[8]
Bart et al. (2021)	Sweat EVs	*miR-26a-5p*, *miR-200c-3p*, *let-7a*, *miR-148a-3p*, *miR-193-3p*, *miR-21-5p*, *miR-24-3p*	Vigorous exercise	Sweat was a potential non-invasive biomarker source for NA-based biomarkers	Biomarker discovery; early human/pilot study	13 donors for initial screen; RNA collection from 25 individuals (20 samples used in analysis)	[5]

Abbreviations: AD, atopic dermatitis; AUC, area under the curve; DF, diabetic foot; DM, diabetes mellitus; EV, extracellular vesicle; FFPE, formalin-fixed, paraffin-embedded; ISF, interstitial fluid; NA, not applicable; PASI, Psoriasis Area and Severity Index. * denotes the miRNA passenger strand (the complementary strand generated from the precursor miRNA duplex); this notation is generally retained to reflect the nomenclature used in the original study.

**Table 2:** Skin-derived lncRNAs as potential biomarkers in various dermatological diseases.

Authors (Year)	Sample	Biomarker(s)	Disease	Clinically Relevant Findings	Cohort Size	Intended Use; Evidence Level	Ref
Gerami et al. (2014)	Skin	*LINC00518*	Melanoma	LINC00518 and/or PRAME differentiated melanoma from nonmelanoma lesions with 91% sensitivity (95% CI: 83–96) and 69% specificity (95% CI: 64–74) in an independent validation cohort; negative predictive value > 99%	555 pigmented lesions (157 training, 398 validation samples); validation cohort: 87 melanomas, 311 nonmelanoma lesions	Diagnostic; independent external validation	[85]
Ferris et al. (2018)	Skin	*LINC00518*	Melanoma	PLA incorporating *LINC00518* achieved 95% sensitivity, 91% specificity, and >99% NPV in a clinical test. Amongst PLA-positive lesions, 37% were confirmed as melanoma, whilst 93% of lesions positive for both *LINC00518* and *PRAME* were confirmed as melanoma.	381 patients (51 PLA-positive, 330 PLA-negative); of the 51 positive cases, 19 were confirmed melanomas	Diagnostic; clinical validation	[84]
Liu et al. (2021)	Skin	*LINC00518*	Melanoma radioresistance	*LINC00518* is greatly upregulated in melanoma and associated with poor overall survival (OS) and relapse-free survival (RFS); potential therapeutic target	Validation cohort: 12 paired CMM and normal tissues; public datasets: 73 melanoma and 17 normal samples; survival analysis: 436 melanoma patients	Prognostic/therapeutic; internal validation and mechanistic evidence	[88]
Wu et al. (2024)	Skin	*AC008687.3*, *AC009495.1*, *AC245128.3*, *AL512363.1*, *LINC00518*, *LINC02416*, *MCCC1-AS1*	Melanoma	Prognostic stratification; nomogram AUCs 0.821, 0.795, 0.820 at 3, 5, 10 years	408 melanoma patients for prognostic analysis	Prognostic; biomarker stratification	[86]
Chen et al. (2022)	Skin	*HOTAIR*	Cutaneous squamous carcinoma	*HOTAIR* significantly upregulated in CSCC, and high expression linked to worse overall survival, suggesting its potential as a prognostic marker and therapeutic target	60 paired clinical samples: 32 non-recurrent and 28 recurrent CSCC cases	Prognostic; mechanistic evidence	[91]
Yu et al. (2024)	Skin	*THOC7-AS1*	Cutaneous squamous carcinoma	*THOC7-AS1* expression correlated with adverse clinicopathological features via *FSTL1*	66 patients	Prognostic; mechanistic evidence	[92]

Abbreviations: AUC, area under the curve; CMM, cutaneous malignant melanoma; CSCC, cutaneous squamous cell carcinoma; NPV, negative predictive value; OS, overall survival; PLA, Pigmented Lesion Assay; RFS, relapse-free survival.

## Data Availability

No new data were created or analyzed in this study. Data sharing is not applicable to this article.

## Abbreviations

Cell-free DNA, cfDNA; cell-free nucleic acid, cfNA; circulating tumour DNA, ctDNA; circular RNA, circRNA; deoxyribonucleic acid, DNA; extracellular RNA, exRNA; extracellular vesicle, EV; high-density lipoprotein, HDL; hypoxia-inducible factor 1 alpha, HIF-1α; interleukin, IL; interstitial fluid, ISF; Janus kinase/signal transducer and activator of transcription, JAK/STAT; long non-coding RNA, lncRNA; metastasis-associated lung adenocarcinoma transcript 1, MALAT1; messenger RNA, mRNA; microRNA, miRNA; mitochondrial DNA, mtDNA; non-coding RNA, ncRNA; next-generation sequencing, NGS; nuclear factor kappa B, NF-κB; nucleotides, nt; PIWI-interacting RNA, piRNA; Psoriasis Area and Severity Index, PASI; quantitative polymerase chain reaction, qPCR; RNA-induced silencing complex, RISC; RNA sequencing, RNA-seq; small nucleolar RNA, snoRNA; small nuclear RNA, snRNA; Staufen double-stranded RNA-binding protein 1, STAU1; transforming growth factor beta/SMAD signalling pathway, TGF-β/SMAD.
